# Serum proteome profiling of plateau acclimatization in men using Olink proteomics approach

**DOI:** 10.14814/phy2.70091

**Published:** 2024-12-26

**Authors:** Jingyu Pan, Yue Dong, Zhihao Zou, Tianyan Gu, Ling Chen, Kai Li, Li Wang, Qinghai Shi

**Affiliations:** ^1^ Medical College of Shihezi University Urumqi Xinjiang China; ^2^ Clinical Laboratory Diagnostic Center General Hospital of Xinjiang Military Command Urumqi Xinjiang China; ^3^ The Second Department of Cadre Health Care Chinese Medicine Hospital Affiliated to Xinjiang Medical University Urumqi Xinjiang China; ^4^ Department of Neurosurgery General Hospital of Xinjiang Military Command Urumqi Xinjiang China; ^5^ Graduate School of Xinjiang Medical University Urumqi Xinjiang China

**Keywords:** hypoxia, metabolism, Olink, plateau acclimatization, proteomics

## Abstract

Plateau acclimatization involves adaptive changes in the body's neurohumoral regulation and metabolic processes due to hypoxic conditions at high altitudes. This study utilizes Olink targeted proteomics to analyze serum protein expression differences in Han Chinese individuals acclimatized for 6 months–1 year at 4500 and 5300 m altitudes, compared to those residing at sea level. The objective is to elucidate the proteins' roles in tissue and cellular adaptation to hypoxia. We identified 54 metabolism‐related differentially expressed proteins (DEPs) in the serum of the high‐altitude group versus the sea‐level group, comprising 20 significantly upregulated and 34 downregulated proteins. Notably, 2 proteins were upregulated and 11 downregulated at both 4500 and 5300 m altitudes. The top three protein correlations among DEPs included CRKL with CA13, RNASE3 with NADK, and NADK with APEX1, alongside APLP1 with CTSH, CTSH with SOST, and CTSH with NT‐proBNP in inverse correlations. KEGG enrichment analysis indicated significant DEP involvement in various metabolic pathways, particularly those associated with hypoxic cellular metabolism like glycolysis/gluconeogenesis and the HIF‐1 signaling pathway. Correlation with clinical phenotypes showed positive associations of SOST, RNASE3, CA13, NADK, and CRKL with SaO_2_ and negative correlations with Hemoglobin and Hematocrit; ALDH1A1 positively correlated with Triglyceride; and SDC4 inversely correlated with Uric acid levels. This study provides insights into specific DEPs linked to metabolic adaptations in high‐altitude acclimatized individuals, offering a foundation for understanding acclimatization mechanisms and potential therapeutic targets.

## 1 INTRODUCTION

Globally, more than 140 million people permanently reside above 2500 m above sea level (Gudbjartsson et al., 2019). Concurrently, an increasing number of individuals are relocating to high‐altitude regions for employment or tourism. These plateau regions are primarily characterized by their low atmospheric pressure and oxygen content, extreme cold, dryness, and intense ultraviolet radiation exposure. Among these factors, hypoxia, or low oxygen levels, presents the most significant challenge to human activities, with its impact intensifying as altitude increases (Burtscher et al., 2021, 2022). In response to prolonged exposure to this low‐oxygen plateau environment, organisms initiate a series of compensatory and acclimatized changes at a holistic level. This process, known as plateau acclimatization, involves non‐genetic variations that enable the body to adjust to the low oxygen and cold conditions, transitioning the body's internal environment from imbalance to balance, and ultimately achieving harmony between the internal and external environments (Wang et al., 2012). The acclimatization process to the plateau environment is time‐dependent and gradual, with most individuals capable of achieving satisfactory acclimatization. However, this process is influenced by the method of ascent, duration at altitude, and the altitude itself. Rapid ascension typically results in poor acclimatization, whereas a gradual and stepped entry into the plateau is more conducive to effective acclimatization, with longer plateau residence leading to more stable adaptation (Li, Zhang, & Zhang, 2018). Plateau acclimatization encompasses changes in the neurohumoral regulatory system, as well as physiological and biochemical metabolic processes, during the hypoxic phase at high altitudes (Zhang et al., 2022). Previous studies have predominantly focused on the acclimatized metabolic alterations in the hematopoietic system induced by hypoxia. These include the activation of hypoxia‐inducible factors, shifts in anaerobic glycolysis and lipid metabolism, and variations in the inflammatory response at the tissue and cellular level (Li, Li, et al., 2018). In addition, the examination of a broad spectrum of end products or intermediates of various tissues and cellular metabolisms in the circulatory system in peripheral blood can provide extensive information on hypoxia‐acclimatized metabolic changes. Among these, the expression changes of numerous end products or intermediates in the circulatory system's peripheral blood are particularly informative, although the detection and analysis of trace and tiny protein molecules have been challenging. This difficulty arises due to these molecules being easily masked by the high abundance of proteins in the blood, thus creating bottlenecks in related research.

Proteins in peripheral blood, originating from various organs or cells, can be transferred between mother and child through the placenta (Pernemalm et al., 2019; Suhre et al., 2021). These proteins play pivotal roles in numerous biological processes, such as signaling, transport, growth, repair, and defense against infections (Sun et al., 2018). Abnormal protein expression in plasma may indicate upstream DNA or RNA molecule anomalies and the influence of external stimuli (Mesleh et al., 2021). Blood samples, owing to their minimally invasive nature, are extensively utilized in diagnostics and research (Connelly et al., 2020). Recent advancements in proteomics technologies, including liquid chromatography–tandem mass spectrometry (LC–MS/MS) (Maekawa & Mano, 2022), protein microarrays (Zhu & Snyder, 2003), and Olink‐targeted proteomics (Carlyle et al., 2022), have primarily focused on identifying novel biomarkers and potential therapeutic targets. These technologies enable high‐coverage qualitative and quantitative protein analysis, offering diverse applications in disease diagnosis, drug discovery, and pathogenic mechanism research. Olink‐targeted proteomics, in particular, is widely employed in basic and clinical studies of various diseases due to its femtogram‐level sensitivity and capability to detect low‐abundance proteins. This study aims to utilize Olink‐targeted proteomics to examine the differential expression of small molecule proteins related to cellular metabolism in the serum of plateau well‐acclimatized populations compared to plain populations. The objective is to analyze their role in regulating cellular hypoxia metabolism, thereby providing a reference for understanding the mechanisms of plateau acclimatization and identifying new drug targets to enhance acclimatization.

## 2 METHODS

### 2.1 Subjects

**FIGURE 1:**
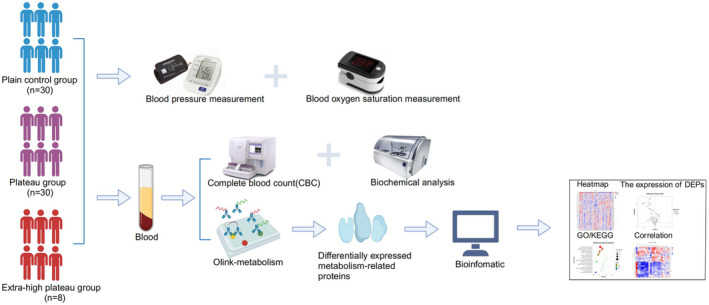
Serum olink‐targeted proteomics study strategy and schematic. Recruitment of subjects from high altitude group, extra‐high altitude group, and plain control group. Parameters measured include blood pressure, heart rate, and fingertip arterial SaO_2_. Venous blood samples collected for complete blood count (CBC) and biochemical analysis. Application of Olink‐targeted proteomics for detection of serum differentially expressed proteins (DEPs) associated with cellular metabolism, accompanied by bioinformatics analysis.

This study, conducted between December 2020 and February 2023, included 38 subjects acclimatized to the Tibetan plateau environment. The cohort comprised 30 subjects residing at 4500 m in the plateau group and eight subjects residing at 5300 m in the extra‐high plateau group. Additionally, 30 healthy subjects residing long‐term in the plains region of Xinjiang Urumqi served as the plains control group. The inclusion criteria for the plateau group were: (1) male individuals, originally from plains regions below 1000 m, who migrated to an average altitude of 4500 m or 5300 m, aged between 18 and 28 years, with a continuous residence time of 6–12 months; (2) those well‐acclimatized to plateau life without current illness; and (3) no prior exposure to altitudes exceeding 2000 m and no history of cardiac, pulmonary, or other underlying health conditions. The plains control group inclusion criteria included: (1) male individuals, who have been lifelong residents at altitudes below 1000 m, without any high‐altitude travel exceeding 2000 m in the past 6 months; (2) absence of chronic diseases and normal liver or renal function, electrocardiograms, chest radiographs, abdominal ultrasound, and other clinical indicators. Exclusion criteria encompassed: (1) individuals with primary or secondary erythrocytosis; and (2) recent history of trauma, surgery, fever, inflammation, or conditions that could influence test results, such as poor physical condition, alcohol consumption, and chronic medication use. Informed consent was obtained from all participants, in compliance with the Medical Ethics Committee guidelines of the General Hospital of the Xinjiang Military Command. The study design and experimental procedures are detailed in Figure 1.

### 2.2 Collection of basic information and physiological indicators

Data on the age, height, and weight of the subjects were systematically collected. In a resting state, heart rate and fingertip arterial oxygen saturation (SaO_2_) measurements were conducted using a finger‐clip pulse oximeter (Jiangsu Yuyue YX307). Additionally, systolic and diastolic blood pressure readings were obtained using an upper arm electronic sphygmomanometer (Dalian Omron HEM‐7127 J).

### 2.3 Sample preparation

To control for diet‐induced variations, all participants in the study fasted for 12 h. The following morning, 5 mL of blood was drawn from the elbow vein into a separation gel vacuum tube and 2 mL into an EDTA‐K2 anticoagulation tube. Blood samples from the separation gel vacuum tubes were processed within 2 h: they were centrifuged at 4°C at 3000 rpm for 10 min, after which the upper serum layer was collected. This serum was partially used for biochemical index analysis, while the remainder was stored at −80°C for proteomics analysis. For the plateau and extra‐high plateau group, CBC and serum biochemical analyses were conducted using a BC5800 blood analyzer (Shenzhen Mindray Bio‐Medical Corporation, China) and a BS2000 biochemical analyzer (Shenzhen Mindray Bio‐Medical Corporation, China), respectively, along with their associated reagents. The plain control group underwent CBC testing with an XE‐5000 blood analyzer (Sysmex Corporation, Japan) and serum biochemical analysis with a Cobas C701 biochemical analyzer (Roche Corporation, Switzerland), using their respective reagents. CBC test parameters included red blood cell count (RBC), hemoglobin (Hb), and hematocrit (HCT). Biochemical analysis parameters encompassed total protein (TP), albumin (ALB), urea, uric acid (UA), triglycerides (TG), total cholesterol (TC), high‐density lipoprotein (HDL), low‐density lipoprotein (LDL), alanine aminotransferase (ALT), aspartate aminotransferase (AST), total bilirubin (TBILI), direct bilirubin (DBILI), and lactate dehydrogenase (LDH).

### 2.4 Proteomics testing

The Olink Target 92 Metabolism Reagent Kit (Olink Proteomics AB, Uppsala, Sweden), provided by Hangzhou Lianchuan Biotechnology Co., was employed to detect the specified targets (refer to Table S1). The fundamental principle of Olink targeted proteomics technology involves utilizing a pair of DNA oligonucleotide‐labeled antibodies, complementary and capable of hybridization, that simultaneously bind to a target protein. The protein under investigation brings these antibodies into close proximity, allowing the paired DNA oligonucleotides on the antibodies to form a double‐stranded DNA tag via a hybridization reaction. This double‐stranded DNA tag is then quantitatively analyzed using microfluidic qPCR, with the quantity of amplicons reflecting the initial concentration of the target protein. The essential procedural steps included sample addition, hybridization incubation, extension, and amplification, microarray pre‐processing, and on‐boarding, followed by data analysis. Quality control of the resulting cyclic count threshold (Ct) data was conducted using three negative controls for limit of detection (LOD) calculation and three interplate controls (IPC) containing group 92 antibodies. The measured values were expressed as log2‐transformed values of normalized protein expression (NPX), calculated using the formula: Target dCt = Target Ct + Negative Control Ct + IPC Ct; Target ddCt = Correction Factor (reagent batch variable) + Target dCt. Data analysis was performed using Olink NPX Signature 1.5.3.0 software. Differential expression of proteins was determined based on a *p*‐value of <0.05 in the comparison of two NPX mean t‐tests, with values >0 indicating up‐regulation and <0 indicating down‐regulation.

### 2.5 Analysis of differentially expressed proteins

Differentially expressed proteins (DEPs) among the three study groups were analyzed using the ‘Olink®Analyze’ package in R. Proteins exhibiting a *p*‐value of less than 0.05 were considered significantly differentially expressed between groups. Heatmaps and volcano plots illustrating the distribution of DEPs were generated using the ‘ggplot2’ package in R.

### 2.6 Gene ontology and kyoto encyclopedia of genes and genomes enrichment analysis

Gene ontology (GO) and kyoto encyclopedia of genes and genomes (KEGG) (Kanehisa & Goto, 2000); (Kanehisa, 2019) enrichment analyses were conducted using the ‘ggplot2’ package in R. In these analyses, all significantly DEPs were mapped to corresponding terms or pathways in the GO and KEGG databases. The identification of GO terms or KEGG pathways significantly enriched with DEPs, compared to a specified background, was achieved through hypergeometric tests. The top 20 GO entries and KEGG pathways were then visually represented in scatter and bar plots.

### 2.7 Correlation analysis

Pearson correlation analysis was utilized to evaluate the relationship between the expression levels of the DEPs. Heatmaps were generated to depict the distribution of correlation clusters, while scatter plots were employed to illustrate the strength of these correlations. Additionally, Pearson correlation analysis was applied to investigate the association between the DEPs and the clinical characteristics of the study participants. The correlation coefficient, approaching a value of 1, indicates a stronger relationship between two variables. The statistical significance of the correlation coefficients was ascertained using a correlation coefficient *p*‐value calculator.

### 2.8 Statistical analyses

Statistical analysis of the data was conducted using SPSS software version 26.0. Variables following a normal distribution were expressed as Mean ± Standard Deviation (Mean ± SD). Group comparisons were performed using one‐way ANOVA, with Bonferroni correction applied for pairwise comparisons. Non‐normally distributed measures were presented as medians with interquartile ranges (*M* [*P*25, *P*75]). Comparisons between groups for these measures were executed using the Kruskal–Wallis *H*‐test, again with Bonferroni correction for pairwise comparisons. In the proteomics section, statistical analyses were performed utilizing the ‘Olink®Analyze’ package in R software (version 2.0.0). Two‐sided tests were executed, and differences were deemed statistically significant at a *p*‐value of less than 0.05.

### 2.9 Ethics approval and consent to participate

The protocol, which adhered to the principles of the Declaration of Helsinki, received approval from the Ethics Committee of the General Hospital of Xinjiang Military Command. All subjects provided their written informed consent.

## 3 RESULTS

### 3.1 Basic information and analysis of physiological and hematological indicators

All subjects in the three groups were male to exclude the influence of gender. The lifestyles and diets of Han Chinese subjects in the plateau and extra‐high plateau groups were similar, supplied through the same safeguarded pathways. The general characteristics and blood indices of the subjects are outlined in Table 1. There were no significant differences observed in height, BMI, or systolic blood pressure across the three groups. When comparing age, a slight difference was noted, with subjects in the extra‐high plateau group being marginally older, though the age difference was not substantial. In terms of physiological indices such as diastolic blood pressure, heart rate, and SaO_2_, there were significant disparities among the groups (*p* < 0.001). The CBC results revealed notable differences in RBC, Hb, and HCT counts (*p* < 0.001), which align with typical physiological adaptations to high‐altitude environments. As altitude increased, there was a corresponding rise in both RBC and Hb levels. Blood biochemical indices including UA, TBILI, DBILI, TP, ALB, GLOB, TC, LDL, HDL, AST, LDH, Urea, and TG showed statistical differences between the groups (*p* < 0.05). These variations suggest distinct metabolic processes in protein, fat, purine, bilirubin, and hepatic enzymes among individuals who migrate to high altitudes compared to those in plains populations.

**TABLE 1:** Clinical characteristics of the participants for the Olink experiment.

	Plain control group (*n* = 30)	Plateau group (*n* = 30)	Extra‐high plateau group (*n* = 8)	F/Z	*p‐*value
Age, year	22.03 ± 1.56	21.80 ± 1.99	26.12 ± 1.36*#	20.587	<0.001
Height, cm	172.77 ± 4.66	175.80 ± 5.74	176.12 ± 6.08	2.853	0.065
BMI, kg/m2	22.04 ± 1.52	22.11 ± 2.22	23.41 ± 2.34	1.640	0.202
Systolic blood pressure, mmHg	115.50 (110.25–124.75)	118.00 (112.00–124.00)	111.50 (107.50–115.50)	2.72	0.257
Diastolic blood pressure, mmHg	69.07 ± 6.81	76.67 ± 9.43*	76.25 ± 6.23	7.34	0.001
Heart rate, times/min	72.80 ± 11.84	85.10 ± 14.48*	83.88 ± 11.12	7.201	0.001
SaO_2_, %	98.83 ± 0.38	86.93 ± 4.86*	84.25 ± 3.06*	114.307	<0.001
Hemoglobin (Hb), g/L	155.77 ± 10.02	201.03 ± 16.59*	223.0 ± 25.43*#	94.385	<0.001
Hematocrit (HCT), %	46.97 ± 2.66	63.46 ± 5.70*	62.61 ± 8.11*	90.279	<0.001
Erythrocyte count (RBC), ×1012/L	5.20 ± 0.33	6.21 ± 0.62*	6.87 ± 1.03*#	37.157	<0.001
Uric acid(UA), μmol/L	315.13 ± 45.95	314.14 ± 58.41	526.62 ± 127.31*#	37.682	<0.001
Total bilirubin(TBILI), μmol/L	13.55 (10.30–17.20)	20.95 (16.83–31.12)*	21.00 (17.90–30.65)*	24.698	<0.001
Direct bilirubin, (DBILI), μmol/L	4.75 (3.62–6.30)	6.85 (5.67–9.47)*	9.55 (7.78–11.80)*	17.036	<0.001
Glutamate‐pyruvate transaminase(ALT), U/L	17.00 (12.00–22.75)	17.70 (15.00–25.43)	16.00 (11.75–27.25)	0.738	0.691
Glutamic oxaloacetic transaminase(AST), U/L	19.00 (16.25–20.00)	26.00 (23.00–30.00)*	24.50 (16.75–28.75)	28.848	<0.001
Lactate dehydrogenase(LDH), U/L	161.73 ± 23.37	283.37 ± 75.82*	204.50 ± 91.28#	30.251	<0.001
Total Protein(TP), g/L	72.38 ± 3.68	74.33 ± 3.60	77.46 ± 5.31*	5.924	0.004
Albumin(ALB), g/L	47.34 ± 1.97	46.58 ± 1.64	49.12 ± 3.88#	4.589	0.014
Globulin(GLOB), g/L	25.04 ± 2.81	27.75 ± 3.13*	28.32 ± 2.71*	7.835	<0.001
Urea, mmol/L	4.77 ± 1.19	5.88 ± 1.07	6.37 ± 5.26	3.162	0.049
Total cholesterol(TC), mmol/L	3.80 ± 0.67	4.29 ± 0.82*	4.52 ± 0.94	4.481	0.015
Triglyceride(TG), mmol/L	0.82 ± 0.29	1.22 ± 0.41*	1.96 ± 1.20*#	16.185	<0.001
Low Density Lipoprotein(LDL), mmol/L	1.88 ± 0.40	2.23 ± 0.50*	2.81 ± 0.81*#	11.623	<0.001
High density lipoprotein(HDL), mmol/L	1.06 ± 0.23	1.50 ± 0.27*	1.20 ± 0.21#	24.747	<0.001

*Note*: For two‐by‐two comparisons, *p < 0.05 compared with plains control group; #*p* < 0.05 compared with plateau group.

### 3.2 Analysis of serum metabolism‐related DEPs

**FIGURE 2:**
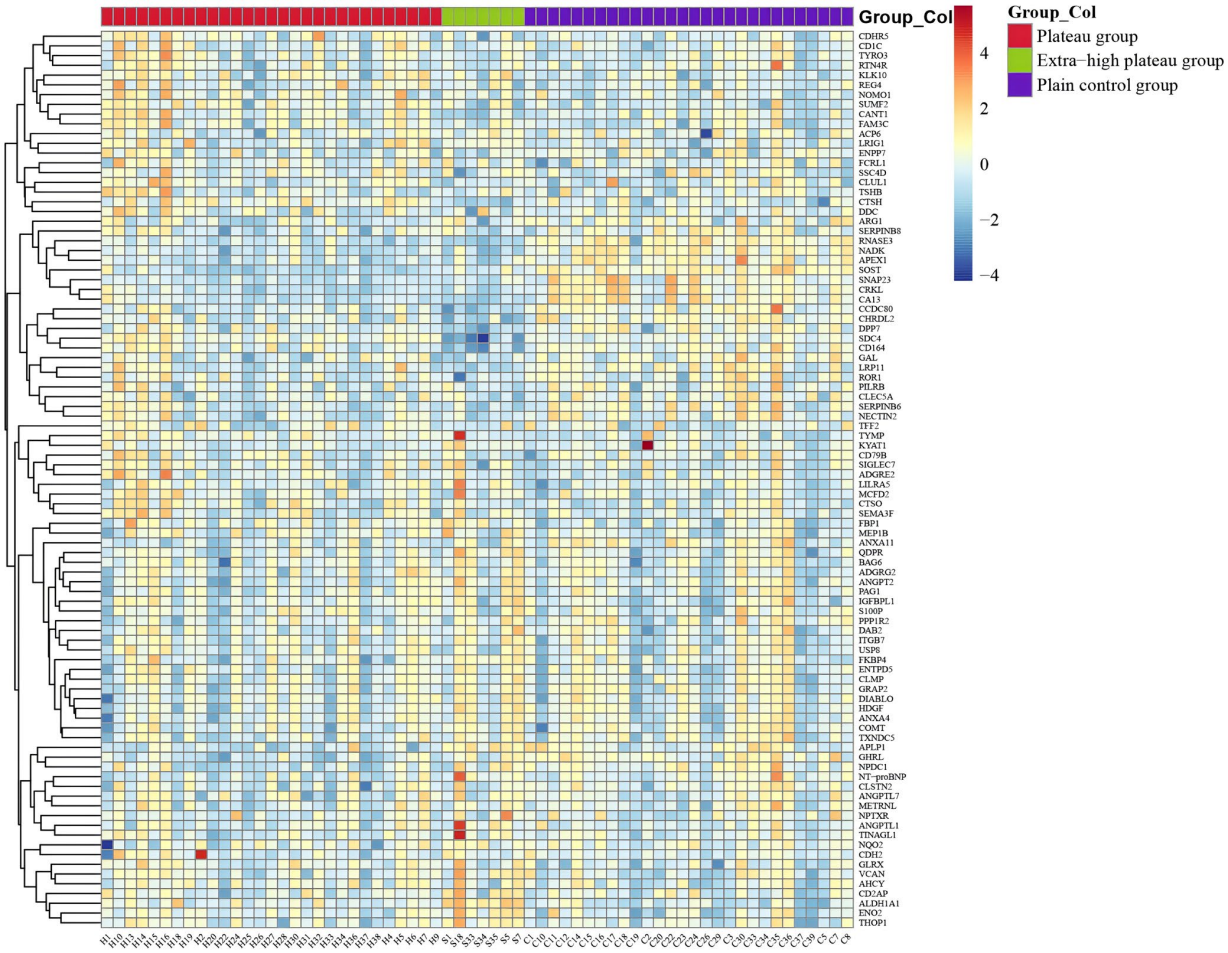
Analysis of differential expression of 92 metabolism‐related target proteins in serum from plateau, extra‐high plateau, and plain control groups. Heatmap depicting clustering analysis of serum differentially expressed proteins (DEPs) in subjects from plateau, extra‐high plateau, and plain control groups.

**FIGURE 3:**
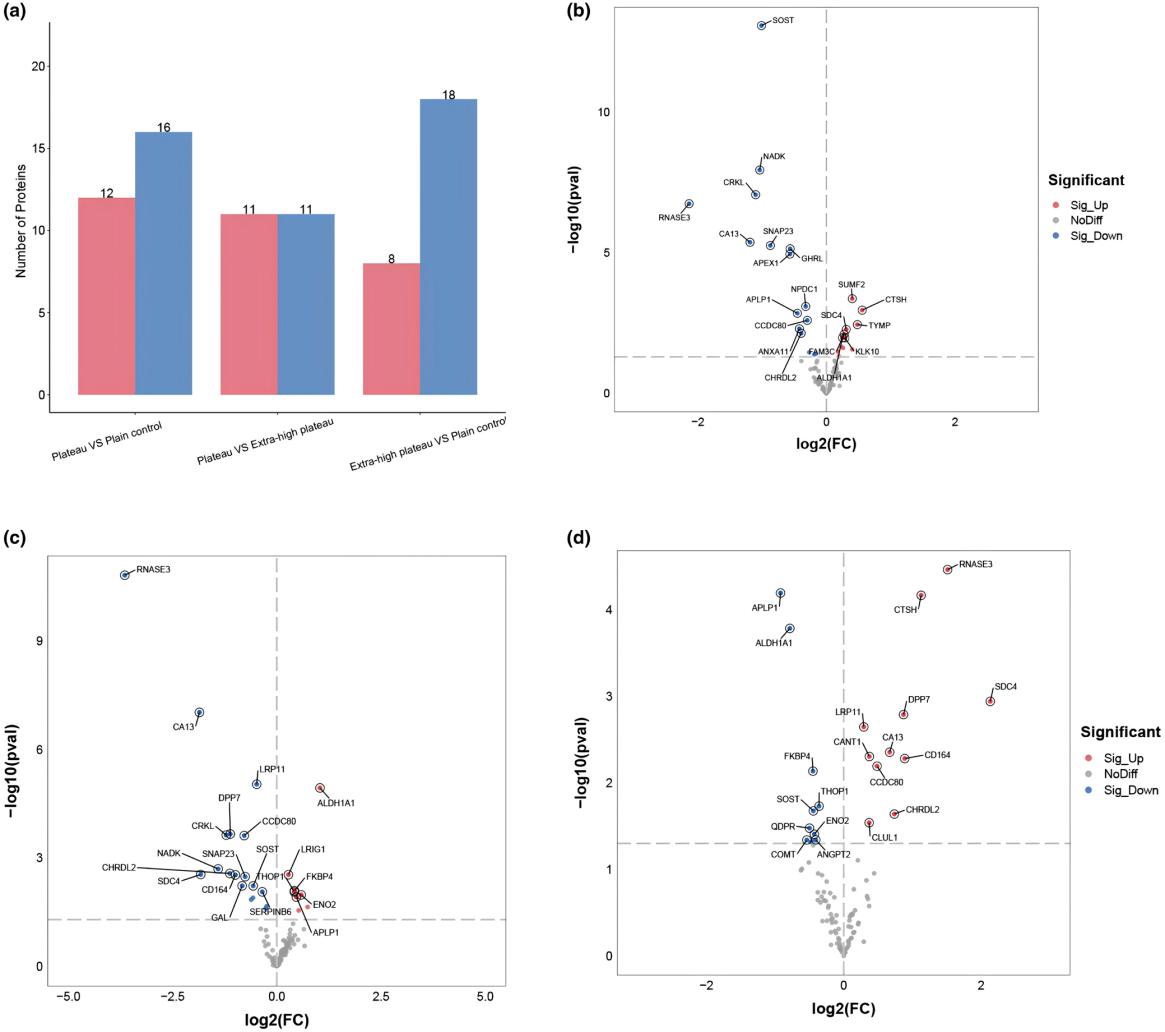
Analysis of differential expression of 92 metabolism‐related target proteins in serum from plateau, extra‐high plateau, and plain control groups. (a) Comparative analysis between plateau, extra‐high plateau, and plain control groups, with a histogram indicating the count of DEPs. (b) Volcano plot illustrating the distribution of DEPs in the plateau group versus the plain control group. (c) Volcano plot showing DEP distribution when comparing the plateau group with the plain control group. (d) Volcano plot representing DEP distribution in the plateau group compared with the extra‐high plateau group. In the heatmap, each *x*‐axis coordinate represents a sample, each *y*‐axis coordinate signifies a target protein, and the color bar's gradient from blue to red indicates expression levels; darker blue signifies lower expression, darker red indicates higher expression. In the volcano plots, the horizontal axis represents the fold change in expressed proteins between the two serum groups [log2 (FC) value], and the vertical axis signifies the statistical significance of the target proteins' differential expression [−log10 (*p*‐value)]. Scattered dots represent different target proteins; gray for proteins with no significant difference, red for upregulated proteins, and blue for downregulated proteins.

**FIGURE 4:**
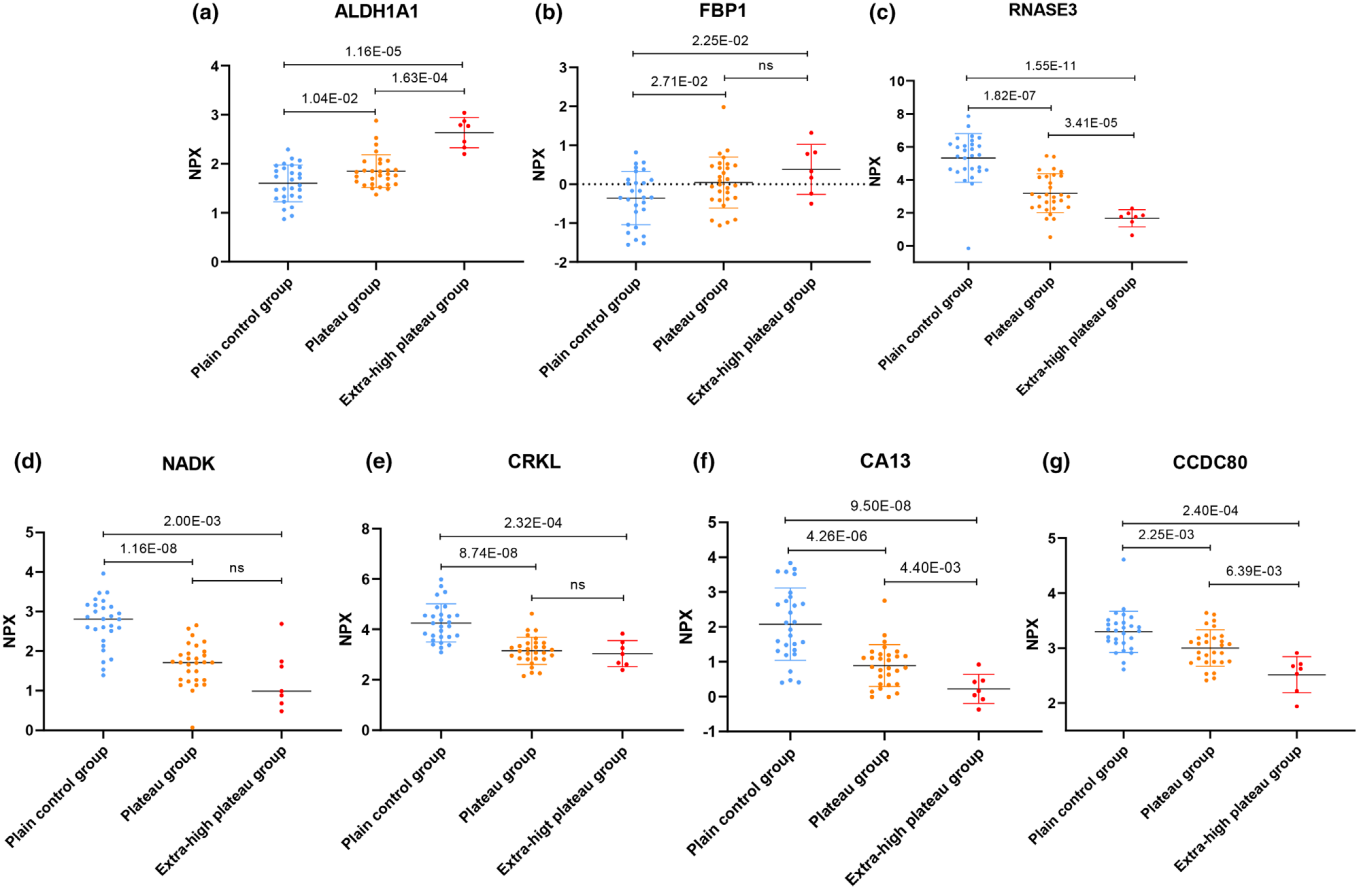
Top 5 DEPs Upregulated or Downregulated with Increasing Altitude and Exhibiting the Smallest *p*‐values in the Serum of Plateau, Extra‐High Plateau, and Plains Control Groups. (a) Upregulation of Retinal Dehydrogenase 1 (ALDH1A1). (b) Upregulation of Fructose‐1,6‐Bisphosphatase 1 (FBP1). (c) Downregulation of Eosinophil Cationic Protein (RNASE3). (d) Downregulation of NAD Kinase (NADK). (e) Downregulation of Crk‐Like Protein (CRKL). (f) Downregulation of Carbonic Anhydrase 13 (CA13). (g) Downregulation of Coiled‐Coil Domain‐Containing Protein 80 (CCDC80). Expression levels normalized to NPX (normalized protein expression).

The expression levels of 92 metabolism‐related target proteins in the serum of subjects from the plateau, extra‐high plateau, and plain control groups were analyzed using Olink targeted proteomics technology. After quality control measures, four samples were excluded due to non‐compliance. The normalized protein expression values (NPX) underwent cluster analysis, as depicted in Figure 2. In comparison to the plain control group, the plateau group exhibited significant up‐regulation in 12 proteins and down‐regulation in 16 proteins, as illustrated in Figure 3a,b and Table 2. The TOP5 DEPs with the smallest *p*‐values in this group included up‐regulated proteins SUMF2, CTSH, TYMP, SDC4, FAM3C, and down‐regulated proteins SOST, NADK, CRKL, RNASE3, CA13. In the extra‐high plateau group, eight proteins were significantly up‐regulated, and 18 were down‐regulated, as shown in Figure 3a,c and Table 3. The TOP5 DEPs in this category were up‐regulated proteins ALDH1A1, LRIG1, FKBP4, THOP1, ENO2, and down‐regulated proteins RNASE3, CA13, LRP11, DPP7, CRKL. When comparing the plateau group with the extra‐high plateau group, 11 proteins were notably up‐regulated, and 11 were down‐regulated, as detailed in Figure 3a,d and Table 4. The TOP5 DEPs with the smallest *p*‐values here included up‐regulated proteins RNASE3, CTSH, SDC4, DPP7, LRP11, and down‐regulated proteins APLP1, ALDH1A1, FKBP4, THOP1, SOST. Statistical analysis of the NPX across the three groups is presented in Table 5, highlighting proteins significantly influenced by increasing altitude. Notably, ALDH1A1 and FBP1 were up‐regulated, while RNASE3, NADK, CRKL, CA13, CCDC80, APEX1, CHRDL2, and LRP11 were down‐regulated. Figure 4a–g provides an analysis of the TOP5 DEPs with the smallest *p*‐values for both up‐regulation and down‐regulation.

**TABLE 2:** Differential expression of metabolic related target proteins in serum of plateau versus plain.

	Protein symbol	Name	Statistic	*p*‐value
Up‐regulation	SUMF2	Sulfatase‐modifying factor 2	3.75	4.28E‐04
	CTSH	Pro‐cathepsin H	3.46	1.10E‐03
	TYMP	Thymidine phosphorylase	3.06	3.56E‐03
	SDC4	Syndecan‐4	2.92	5.27E‐03
	FAM3C	Protein FAM3C	2.76	7.86E‐03
	ALDH1A1	Retinal dehydrogenase 1	2.65	1.04E‐02
	KLK10	Kallikrein‐10	2.65	1.06E‐02
	GLRX	Glutaredoxin‐1	2.38	2.14E‐02
	CDHR5	Cadherin‐related family member 5	2.33	2.41E‐02
	FBP1	Fructose‐1,6‐bisphosphatase 1	2.27	2.71E‐02
	MCFD2	Multiple coagulation factor deficiency protein 2	2.20	3.22E‐02
	CTSO	Cathepsin O	2.03	4.78E‐02
Down‐regulation	SOST	Sclerostin	−9.88	8.67E‐14
	NADK	NAD kinase	−6.74	1.16E‐08
	CRKL	Crk‐like protein	−6.28	8.74E‐08
	RNASE3	Eosinophil cationic protein	−6.02	1.82E‐07
	CA13	Carbonic anhydrase 13	−5.26	4.26E‐06
	SNAP23	Synaptosomal‐associated protein 23	−5.33	5.54E‐06
	GHRL	Appetite‐regulating hormone	−4.96	7.27E‐06
	APEX1	DNA‐(apurinic or apyrimidinic site) lyase	−4.90	1.12E‐05
	NPDC1	Neural proliferation differentiation and control protein 1	−3.55	8.08E‐04
	APLP1	Amyloid‐like protein 1	−3.36	1.41E‐03
	CCDC80	Coiled‐coil domain‐containing protein 80	−3.17	2.52E‐03
	ANXA11	Annexin A11	−2.94	5.00E‐03
	CHRDL2	Chordin‐like protein 2	−2.79	7.24E‐03
	ARG1	Arginase‐1	−2.17	3.44E‐02
	NECTIN2	Nectin‐2	−2.12	3.85E‐02
	LRP11	Low‐density lipoprotein receptor‐related protein 11	−2.09	4.11E‐02

**TABLE 3:** Differential expression of metabolic related target proteins in serum of extra‐high plateau versus plain.

	Protein symbol	Name	Statistic	*p*‐value
Up‐regulation	ALDH1A1	Retinal dehydrogenase 1	7.57	1.16E‐05
	LRIG1	Leucine‐rich repeats and immunoglobulin‐like domains protein 1	3.23	2.88E‐03
	FKBP4	Peptidyl‐prolyl cis‐trans isomerase FKBP4	2.99	7.95E‐03
	THOP1	Thimet oligopeptidase	3.31	8.54E‐03
	ENO2	Gamma‐enolase	3.17	1.04E‐02
	APLP1	Amyloid‐like protein 1	2.89	1.19E‐02
	FBP1	Fructose‐1,6‐bisphosphatase 1	2.71	2.25E‐02
	QDPR	Dihydropteridine reductase	2.63	2.80E‐02
Down‐regulation	RNASE3	Eosinophil cationic protein	−10.71	1.55E‐11
	CA13	Carbonic anhydrase 13	−7.36	9.50E‐08
	LRP11	Low‐density lipoprotein receptor‐related protein 11	−5.64	9.17E‐06
	DPP7	Dipeptidyl peptidase 2	−5.12	2.17E‐04
	CRKL	Crk‐like protein	−4.99	2.32E‐04
	CCDC80	Coiled‐coil domain‐containing protein 80	−5.49	2.40E‐04
	NADK	NAD kinase	−4.49	2.00E‐03
	CHRDL2	Chordin‐like protein 2	−4.36	2.66E‐03
	SDC4	Syndecan‐4	−4.73	2.84E‐03
	CD164	Sialomucin core protein 24	−4.32	2.92E‐03
	SNAP23	Synaptosomal‐associated protein 23	−3.40	3.26E‐03
	GAL	Galanin peptides	−3.33	5.84E‐03
	SOST	Sclerostin	−3.54	5.85E‐03
	SERPINB6	Serpin B6	−3.15	8.54E‐03
	CTSH	Pro‐cathepsin H	−2.84	1.25E‐02
	APEX1	DNA‐(apurinic or apyrimidinic site) lyase	−3.00	1.41E‐02
	NECTIN2	Nectin‐2	−2.61	2.15E‐02
	CANT1	Soluble calcium‐activated nucleotidase 1	−2.68	2.47E‐02

**TABLE 4:** Differential expression of metabolic related target proteins in serum of plateau versus extra‐high plateau.

	Protein symbol	Name	Statistic	*p*‐value
Up‐regulation	RNASE3	Eosinophil cationic protein	5.14	3.41E‐05
	CTSH	Pro‐cathepsin H	6.06	6.76E‐05
	SDC4	Syndecan‐4	5.45	1.14E‐03
	DPP7	Dipeptidyl peptidase 2	4.19	1.62E‐03
	LRP11	Low‐density lipoprotein receptor‐related protein 11	3.43	2.25E‐03
	CA13	Carbonic anhydrase 13	3.45	4.40E‐03
	CANT1	Soluble calcium‐activated nucleotidase 1	3.49	4.94E‐03
	CD164	Sialomucin core protein 24	3.82	5.22E‐03
	CCDC80	Coiled‐coil domain‐containing protein 80	3.51	6.39E‐03
	CHRDL2	Chordin‐like protein 2	2.77	2.29E‐02
	CLUL1	Clusterin‐like protein 1	2.41	2.88E‐02
Down‐regulation	APLP1	Amyloid‐like protein 1	−5.82	6.35E‐05
	ALDH1A1	Retinal dehydrogenase 1	−5.92	1.63E‐04
	FKBP4	Peptidyl‐prolyl cis‐trans isomerase FKBP4	−2.97	7.29E‐03
	THOP1	Thimet oligopeptidase	−2.87	1.85E‐02
	SOST	Sclerostin	−2.77	2.10E‐02
	QDPR	Dihydropteridine reductase	−2.57	3.33E‐02
	ENO2	Gamma‐enolase	−2.53	3.91E‐02
	ANGPT2	Angiopoietin‐2	−2.33	4.55E‐02
	COMT	Catechol O‐methyltransferase	−2.24	4.56E‐02
	GHRL	Appetite‐regulating hormone	−2.31	4.72E‐02
	NQO2	Ribosyldihydronicotinamide dehydrogenase [quinone]	−2.13	4.82E‐02

**TABLE 5:** Differential expression of metabolic related target proteins in serum of plateau versus extra‐high plateau versus plain.

Protein symbol	Name	Statistic	*p*‐value
SDC4	Syndecan‐4	52.68	5.13E‐14
SOST	Sclerostin	49.09	1.97E‐13
RNASE3	Eosinophil cationic protein	32.47	2.50E‐10
NADK	NAD kinase	27.91	2.47E‐09
ALDH1A1	Retinal dehydrogenase 1	24.44	1.61E‐08
CRKL	Crk‐like protein	23.92	2.14E‐08
CA13	Carbonic anhydrase 13	22.63	4.44E‐08
SNAP23	Synaptosomal‐associated protein 23	16.18	2.31E‐06
CCDC80	Coiled‐coil domain‐containing protein 80	15.21	4.36E‐06
CD164	Sialomucin core protein 24	14.28	8.18E‐06
APEX1	DNA‐(apurinic or apyrimidinic site) lyase	13.63	1.28E‐05
CHRDL2	Chordin‐like protein 2	12.82	2.26E‐05
CTSH	Pro‐cathepsin H	12.90	2.12E‐05
GHRL	Appetite‐regulating hormone	12.52	2.79E‐05
APLP1	Amyloid‐like protein 1	12.28	3.31E‐05
DPP7	Dipeptidyl peptidase 2	9.62	2.34E‐04
ENO2	Gamma‐enolase	6.82	2.13E‐03
LRP11	Low‐density lipoprotein receptor‐related protein 11	6.66	2.43E‐03
NPDC1	Neural proliferation differentiation and control protein 1	6.66	2.42E‐03
SUMF2	Sulfatase‐modifying factor 2	6.42	2.96E‐03
THOP1	Thimet oligopeptidase	5.60	5.84E‐03
CANT1	Soluble calcium‐activated nucleotidase 1	5.39	7.01E‐03
QDPR	Dihydropteridine reductase	4.73	1.23E‐02
SERPINB6	Serpin B6	4.70	1.27E‐02
FBP1	Fructose‐1,6‐bisphosphatase 1	4.63	1.34E‐02
TYMP	Thymidine phosphorylase	4.30	1.79E‐02
ANGPTL1	Angiopoietin‐related protein 1	4.16	2.02E‐02
CD2AP	CD2‐associated protein	4.13	2.07E‐02
ANXA11	Annexin A11	4.05	2.23E‐02
NT‐proBNP	–	4.04	2.25E‐02
FAM3C	Protein FAM3C OS=Homo	3.87	2.61E‐02
GAL	Galanin peptides	3.86	2.63E‐02
VCAN	Versican core protein	3.52	3.59E‐02
NECTIN2	Nectin‐2	3.41	3.94E‐02
ANGPT2	Angiopoietin‐2	3.29	4.38E‐02
KLK10	Kallikrein‐10	3.32	4.27E‐02
TINAGL1	Tubulointerstitial nephritis antigen‐like	3.33	4.24E‐02

### 3.3 Correlation analysis between metabolism‐related differentially expressed proteins in serum

**FIGURE 5:**
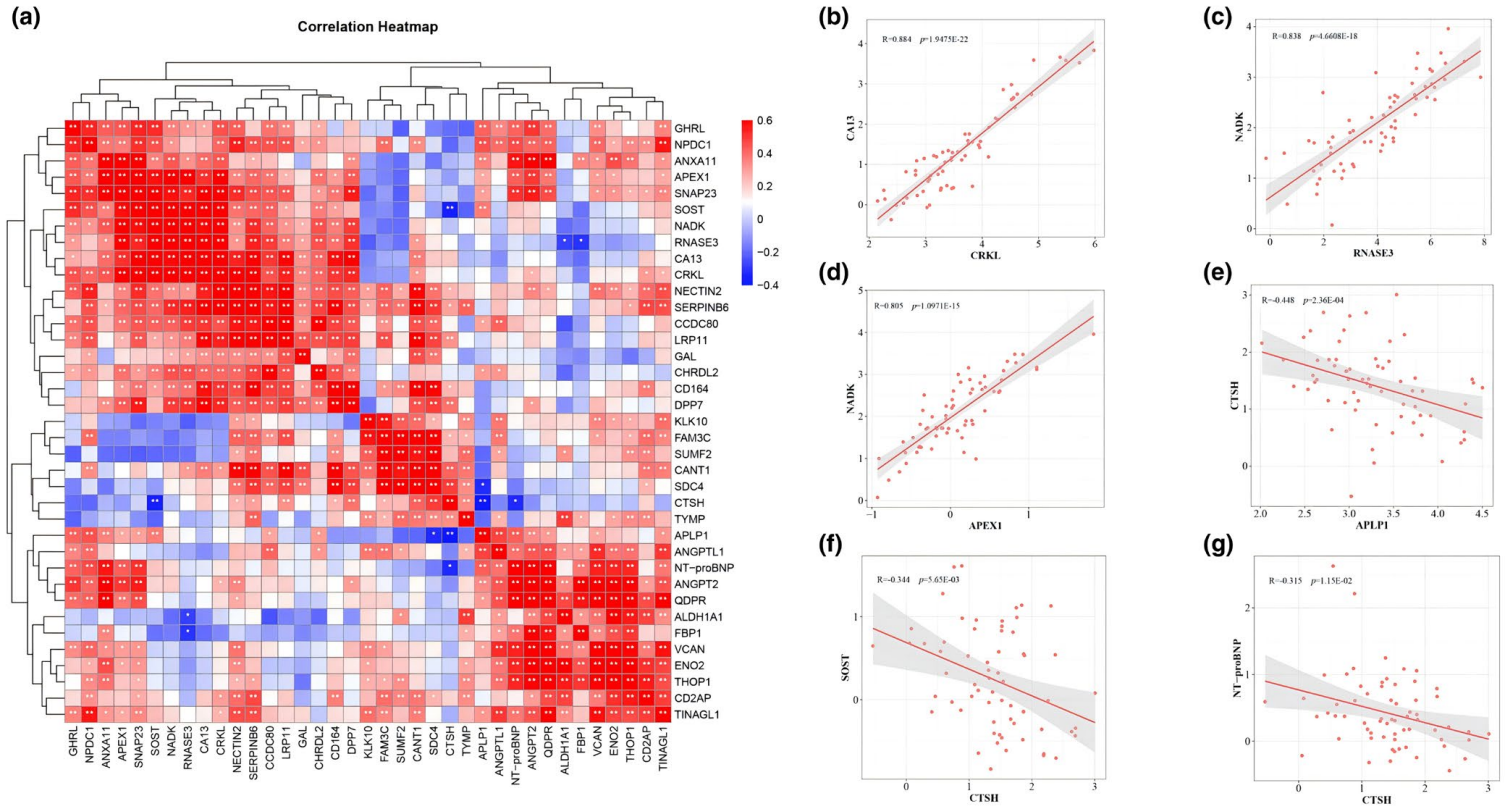
Correlation Analysis of Differentially expressed proteins (DEPs) in the Serum of Plateau, Extra‐High Plateau, and Plains Control Groups. (a) Heatmap representing two‐by‐two comparative correlation analysis of DEPs across the three groups. (b) Positive correlation observed between Crk‐Like Protein (CRKL) and Carbonic Anhydrase 13 (CA13). (c) Positive correlation identified between Eosinophil Cationic Protein (RNASE3) and NAD Kinase (NADK). (d) APEX Nuclease 1 (APEX1) demonstrated positive correlation with NADK. (e) Amyloid Beta Precursor‐Like Protein 1 (APLP1) showed negative correlation with Cathepsin H (CTSH). (f) CTSH exhibited negative correlation with Sclerostin (SOST). (g) Negative correlation between CTSH and N‐terminal pro‐B‐type Natriuretic Peptide (NT‐proBNP) identified. Correlation heatmap denotes positive correlation in red, negative correlation in blue, and absence of correlation in white. ‘R’ represents Pearson's correlation coefficient. Significance levels are indicated as * for *p* < 0.05, ** for *p* < 0.01 and *** for *p* < 0.001.

Considering the potential interrelations and indirect regulatory associations among DEPs associated with plateau acclimatization, a comprehensive correlation analysis of expressed proteins with statistically significant differences was conducted for the three groups, as detailed in Figure 5a and Table S2. The analysis identified 93 pairs of proteins with positive correlations (*R* > 0.5) and 104 pairs with negative correlations (*R* < 0.5). The top three protein combinations demonstrating significant correlations were CRKL and CA13 (*R* = 0.884, *p* = 1.9475E‐22), RNASE3 and NADK (*R* = 0.838, *p* = 4.6608E‐18), and APEX1 and NADK (*R* = 0.805, *p* = 1.0971E‐15), as illustrated in Figure 4b–d. Notable negative correlations included APLP1 with CTSH (*R* = −0.448, *p* = 2.36E‐04), CTSH with SOST (*R* = −0.344, *p* = 5.65E‐03), and CTSH with NT‐proBNP (*R* = −0.315, *p* = 1.15E‐02), as shown in Figure 5e–g.

### 3.4 GO and KEGG enrichment analysis of metabolism‐related DEPs in serum.

**FIGURE 6:**
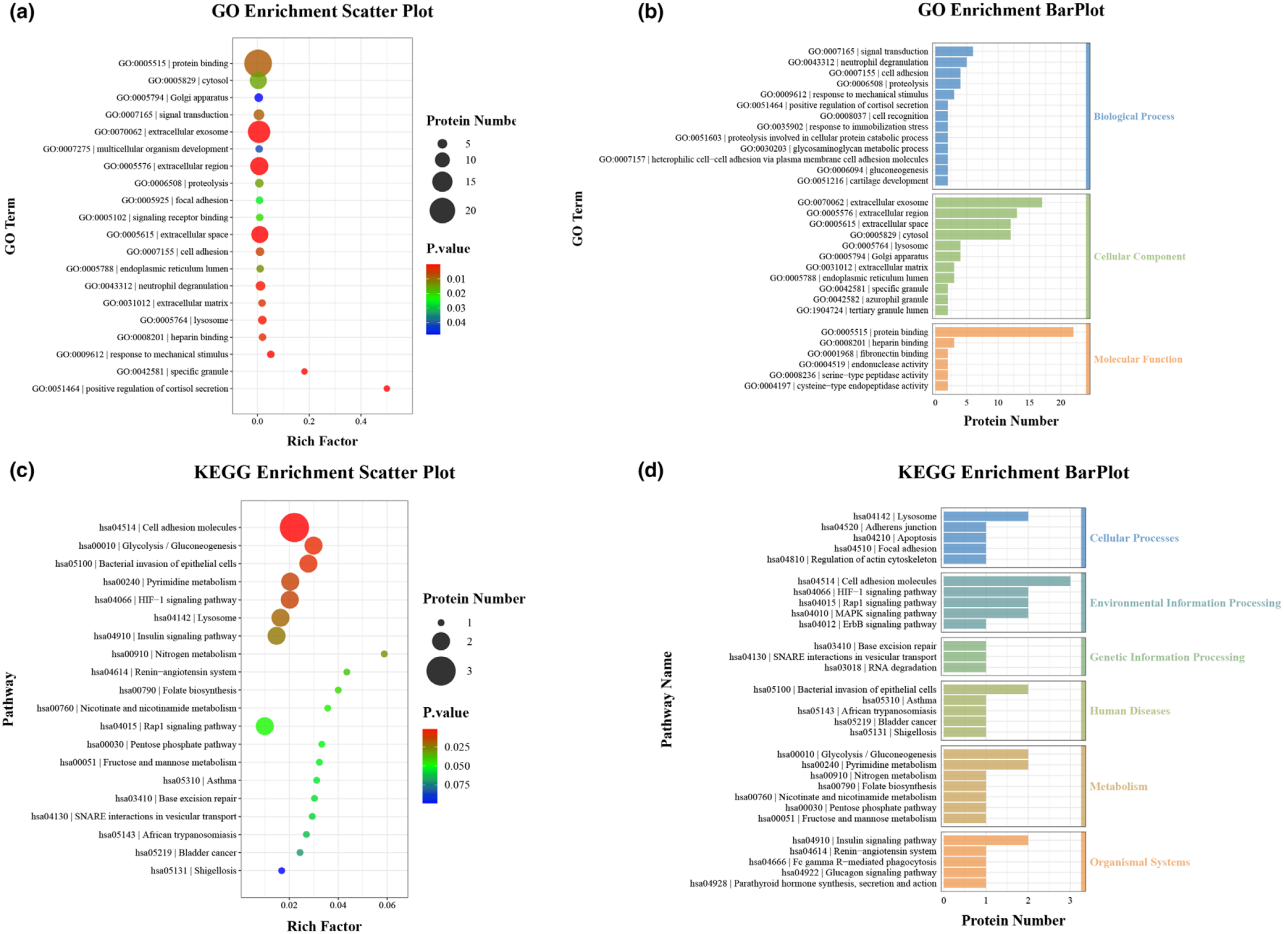
Gene Ontology (GO) and Kyoto Encyclopedia of Genes and Genomes (KEGG) Enrichment Analysis of Differentially Expressed Proteins (DEPs) in the Serum of Plateau, Extra‐High Plateau, and Plains Control Groups. A total of 37 DEPs were analyzed using GO and KEGG enrichment methods. (a) GO enrichment analysis bubble plot illustrating the TOP20 GO terms, selected based on the significance of enrichment (*p*‐value) and the number of enriched proteins. (b) GO enrichment analysis histogram displaying the distribution of DEPs across biological process, cellular component, and molecular function categories based on the number of GO terms enriched. (c) KEGG enrichment analysis bubble plot representing the top 20 pathways, chosen according to the significance of enrichment (*p*‐value) and the number of enriched proteins. (d) KEGG enrichment analysis hierarchical histogram showing the distribution of DEPs across various pathways. In the bubble plots, the Rich factor on the horizontal axis indicates the proportion of DEPs in a specific GO Term relative to the total number of proteins in that term. A higher Rich Factor signifies greater enrichment. The size of the dots corresponds to the number of enriched proteins, and the color denotes the *p*‐value, indicating the significance of the enrichment.

The Olink proteomic metabolism panel was used to examine 92 target proteins, with a focus on the GO classification of 37 proteins that exhibited statistically significant differences among the three comparison groups. The results indicated that the DEPs were predominantly enriched in areas such as protein binding (22 proteins), extracellular exosomes (17 proteins), extracellular region (13 proteins), extracellular space (12 proteins), and cytosol (12 proteins), as shown in Figure 6a,b, and Table S3. Subsequent KEGG enrichment analysis revealed that these DEPs were concentrated in pathways including cell adhesion molecules, glycolysis/gluconeogenesis, bacterial invasion of epithelial cells, pyrimidine metabolism, HIF‐1 signaling pathway, lysosome, insulin signaling pathway, and Rap1 signaling pathway, detailed in Figure 6c,d. Notably, DEPs in glycolysis/gluconeogenesis, such as FBP1 and ENO2, have distinct roles: down‐regulated FBP1 inhibits gluconeogenesis and facilitates glycolysis, whereas up‐regulated ENO2 catalyzes the transformation of 2‐phosphoglyceric acid to phosphoenolpyruvate, serving as a key enzyme in glycolysis. ENO2 and ANGPT2, associated with the HIF‐1 signaling pathway, are regulated by the hypoxia receptor factor HIF‐1. ENO2 is a rate‐limiting enzyme in glycolysis, while ANGPT2 functions as an angiogenic factor. Moreover, some DEPs play crucial roles in nitrogen metabolism, renin‐angiotensin system, folate biosynthesis, and nicotinate and nicotinamide metabolism, as listed in Table S4.

### 3.5 Correlation analysis between metabolism‐related differentially expressed proteins in serum and clinical characteristics of plateau subjects

**FIGURE 7:**
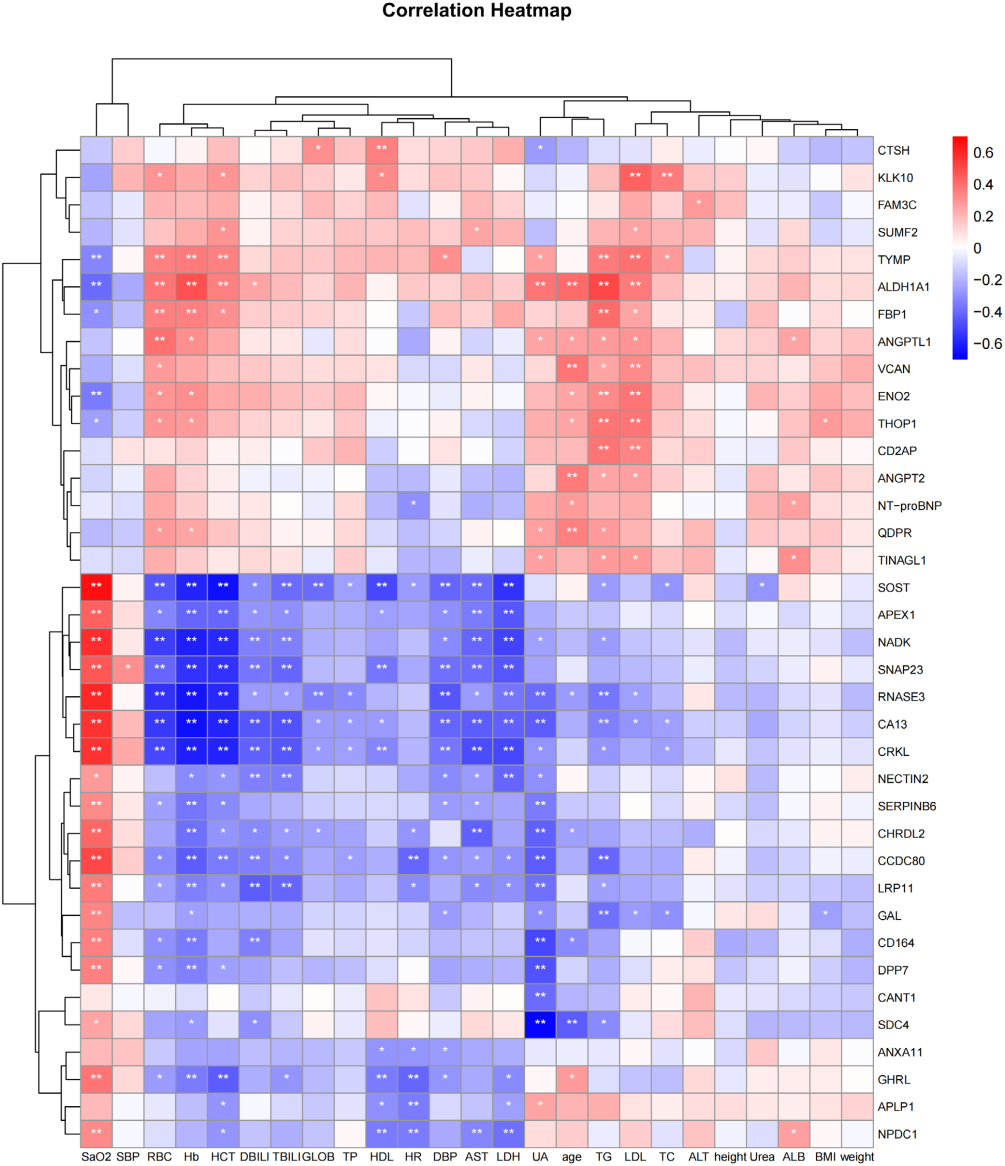
Heatmap of Correlation Analysis Between DEPs in Serum and Clinical Characteristics of Subjects in Plateau, Extra‐High Plateau, and Plain Control Groups. This analysis involved 37 DEPs and their correlation with 4 physiological and 17 blood indices. In the heatmap, the horizontal axis represents clinical characteristic indices, while the vertical axis corresponds to the DEPs. A positive correlation is indicated by red color, a negative correlation by blue color, and no correlation is shown in white. Significance levels are indicated as * for *p* < 0.05, ** for *p* < 0.01 and *** for *p* < 0.001.

Considering the potential correlation between DEPs related to well‐acclimatized metabolism in the plateau‐adapted population and their clinical characteristics, extensive correlation analyses were conducted. These analyses focused on the 37 proteins that showed statistically significant differences among the three groups in relation to four physiological and 17 blood indices (refer to Figure 7 and Table S5). The results revealed seven DEPs with correlation coefficients greater than 0.5 and a positive correlation. Specifically, SOST, RNASE3, NADK, CRKL, CA13, and CCDC80 exhibited a positive correlation with SaO_2_, while ALDH1A1 correlated positively with triglycerides. Conversely, there were seven DEPs with correlation coefficients greater than 0.5 showing negative correlation. Specifically, RNASE3, NADK, CRKL, and CA13 were linked to RBC, HCT, and Hb, while SOST and SNAP23 were only associated with Hb and HCT. Additionally, SDC4 was associated with UA, and both SOST and NADK were associated with LDH.

## 4 DISCUSSION

The plateau environment, characterized by its unique geographical and climatic features, includes low air pressure, reduced oxygen content, significant temperature fluctuations, dry climate, and intense ultraviolet radiation. These factors collectively exert multiple effects on human health. Initial exposure to acute hypoxia at high altitudes can induce plateau reactions such as dyspnea, dizziness, and fatigue, and in severe cases, may lead to conditions like plateau pulmonary edema or cerebral edema (Rodway et al., 2003). In response to extreme plateau environments, the human body activates various mechanisms, leading over time to a new equilibrium. This adaptation involves an initial increase in respiratory rate and depth, cardiac output, heart rate, and blood pressure, followed by an increase in erythrocyte count and anaerobic cellular glycolysis, a process known as plateau acclimatization (Gonzales, 2011). During this acclimatization, physical changes in the blood gradually increase urinary bicarbonate, resulting in a reduction in blood bicarbonate (Zouboules et al., 2018). This stimulates the secretion of erythropoietin (EPO), enhancing the circulatory system's erythrocyte count and oxygen transport capacity, crucial for adapting to high‐altitude oxygen availability (Song et al., 2017). Additionally, increased neovascularization promotes oxygen supply to tissues and cells (Zhou et al., 2023). In this study, we included subjects well‐acclimatized to altitudes of 4500 and 5300 m, residing there for 6–12 months. These subjects exhibited decreased SaO_2_, increased heart rate, and elevated peripheral blood RBC count, hemoglobin concentration, and HCT levels (Deng et al., 2012; Wang et al., 2018; Yao et al., 2022). These characteristics align with those of populations long‐term migrating to high altitudes. Blood biochemical indices also showed differences compared to the plains population, but the results were not entirely consistent with previous studies on migrated plateau populations (Moore, 2017; Ren et al., 2023; Yuan et al., 2023). This discrepancy may be attributed to variations in plateau diets, migration duration, work intensity, and demographic factors such as age and sex. Further research involving a larger observational population and multifactorial multivariate regression analysis is necessary to elucidate these findings.

The prevailing understanding of hypoxic cellular metabolism emphasizes a shift from oxidative phosphorylation to anaerobic glycolysis for energy production (Eaton & Pamenter, 2022; Han et al., 2021; Kietzmann, 2019). Total systemic reliance on anaerobic glycolysis, while aiding acclimatization, can lead to rapid glucose depletion and severe lactic acidosis, impeding effective plateau acclimatization. Midha et al. (2023) noted that during chronic hypoxia, different organs adapt their metabolism uniquely. For example, the heart increases mitochondrial glucose oxidation, while the brain, kidneys, and liver enhance fatty acid uptake to support the TCA cycle. These organ‐specific adaptations result in reduced body fat, decreased blood glucose levels, and improved motor function under hypoxic conditions, highlighting the diverse contributions of organs to whole‐body hypoxic acclimatization and resulting in a catabolic state. Hypoxia triggers a redistribution of cellular fuels. When the body is in a state of overall hypoxia, fuel metabolism adapts through specific pathways to optimize acclimatization. Metabolic products from tissue or cellular hypoxia acclimatization are released into the peripheral blood circulation, some of which regulate target organs via the circulatory system. Others serve as metabolic intermediates or wastes, relying on the circulatory system for removal. These changes in expression and level of product molecules offer insights into metabolic shifts. Lu et al. (2018) observed reduced expression of proteins related to the tricarboxylic acid cycle, ribosomes, metabolic pathways, glyceride metabolism, ascorbate, and hydrochloride metabolism, propionate metabolism, and proteasome in the plasma of a well‐acclimatized plateau population using iTRAQ proteomics. Zhang et al. (2021) explored protein expression differences between chronic plateau disease patients and non‐chronic individuals using iTRAQ 4‐plex proteomics. Enrichment analyses indicated that DEPs were mainly accumulated in peroxisomes, nitrogen metabolism, carbon metabolism, metabolic pathways, purine metabolism, and pentose phosphate pathways. Yang et al. (2022) identified serum differentially expressed protein profiles in well‐ and poorly‐acclimatized individuals after rapid ascent to plateau through Olink proteomics. They found elevated expression of proteins involved in carbohydrate metabolism and those critical in glucose and energy metabolism. The cumulative findings from Midha et al. (2023), Lu et al. (2018), Zhang et al. (2021), and Yang et al. (2022) demonstrate the human body's significant metabolic adaptability during hypoxia. These studies reveal that plateau hypoxia leads to substantial changes in proteins associated with cellular metabolic pathways in the blood, aiding in exploring the metabolic mechanisms of cellular acclimatization to plateau hypoxia and providing novel targets for drugs aimed at preventing high altitude illness or promoting acclimatization.

In plasma, the detection of trace and tiny protein molecules is often challenged by the masking effect of high‐abundance proteins like albumin and immunoglobulin. Their low abundance makes them difficult to detect. However, Olink targeted proteomics represents a highly sensitive and high‐throughput technology, specialized in the targeted detection of specific protein types in blood (or various bodily fluids). Its ability to achieve femtogram‐level sensitivity and efficiently detect low‐abundance proteins constitutes the primary advantage of this technology. In this study, we utilized this technique to assess the expression levels of 92 metabolism‐related target proteins in the peripheral blood of individuals who had migrated to the plateau for 6–12 months and were well‐acclimatized, compared to those from the plains. We identified a total of 37 proteins in the DEP profile. These DEPs may exhibit mutual or indirect regulatory relationships, with 93 pairs showing positive correlation and 104 pairs demonstrating negative correlation. Further Gene Ontology (GO) and Kyoto Encyclopedia of Genes and Genomes (KEGG) enrichment analyses revealed significant enrichment of these DEPs in metabolic pathways such as glycolysis/gluconeogenesis, HIF‐1 signaling pathway, insulin signaling pathway, and the renin‐angiotensin system. These findings suggest that the human body undergoes acclimatized changes from glucose aerobic oxidation to anaerobic glycolysis during plateau hypoxia acclimatization. This includes changes in glucose metabolism hormones, hormones regulating humoral homeostasis, and angiogenesis, where HIF‐1 plays a crucial regulatory role. The results of our enrichment analyses partially align with those reported by Lu et al. (2018), Zhang et al. (2021), and Yang et al. (2022). The differences observed may be attributed to variations in the acclimatization status, duration of stay on the plateau, and differences in altitude among the study populations.

Among the 37 DEPs identified in the three study groups, ALDH1A1 and FBP1 were significantly up‐regulated with increasing altitude, while RNASE3, NADK, CRKL, CA13, CCDC80, APEX1, CHRDL2, and LRP11 showed significant down‐regulation. This suggests that the expression regulation of these DEPs is closely associated with the degree of hypoxia and the extent of cellular metabolic compensation in response to hypoxia. Retinal dehydrogenase 1 (ALDH1A1) plays a crucial role in detoxifying lipid peroxidation byproducts such as acrolein, 4‐hydroxy‐2‐nonenal (4‐HNE), and malondialdehyde (Yoval‐Sanchez & Rodriguez‐Zavala, 2012), and is instrumental in restoring the activity of other aldehyde dehydrogenases and detoxifying enzymes under conditions of increased oxidative stress. The up‐regulation of ALDH1A1 in response to oxidative stress in the plateau environment may provide antioxidant protection. Additionally, a positive correlation was observed between ALDH1A1 and serum triglyceride concentration levels, indicating its potential involvement in triglyceride metabolism. Fructose‐1,6‐bisphosphatase 1 (FBP1), a rate‐limiting glycoheterotrophic enzyme, catalyzes the hydrolysis of fructose‐1,6‐bisphosphate (F‐1,6‐BP) to fructose‐6‐phosphate (F‐6‐P). Obesity and dietary fat intake are known to increase this rate‐limiting enzyme (Choi & Lee, 2023) and a protein phosphatase that inhibits peroxisome proliferator‐activated receptor (PPARα)‐mediated transcription of fatty acid oxidation‐related genes and fatty acid oxidation in mitochondria (Zhang et al., 2023). This is indicative of a link between FBP1 and glycolipid metabolism and fatty acid oxidation, suggesting that abnormal lipid metabolism under plateau hypoxia may influence the expression of this enzyme.

Eosinophil cationic protein (RNASE3), a crucial member of the Ribonuclease A (RNASE A) superfamily, is renowned for its significant role in host immunity. Primarily expressed by leukocytes, RNASE3 exhibits broad‐spectrum antimicrobial activity (Liu et al., 2012). Despite its established role in microbial defense, the function of RNASE3 in cellular hypoxia, particularly in high‐altitude environments, remains less explored and warrants further investigation. NAD kinase (NADK), instrumental in catalyzing the phosphorylation of NAD to generate NADP, plays a pivotal role in various biological functions, including cell metabolism and antioxidant processes (Oka et al., 2023), (Agledal et al., 2010). The high‐altitude plateau environment, characterized by its low‐pressure and low‐oxygen conditions compounded with oxidative stress factors such as intense ultraviolet light, significantly impacts the activity of NADK. Studies by Grose et al. (2006) revealed that the reduction in pyridine nucleotides under ultraviolet irradiation activates NADK, and the resultant increase in NADPH is essential in mitigating cellular oxidative stress, underscoring the enzyme's adaptive response in high‐altitude conditions. Crk‐like protein (CRKL), belonging to the adaptor protein family, is involved in diverse biological functions, such as adhesion, proliferation, migration, and survival (Roy et al., 2020; Song et al., 2019). Research by Zhang et al. (2015) highlighted the protective role of CRKL in hypoxia/reoxygenation (H/R) treated rat H9C2 cardiomyocytes. Their findings indicated that CRKL gene knockdown resulted in reduced cardiomyocyte apoptosis, implicating CRKL as a potential therapeutic target in hypoxia‐induced cardiomyocyte injury. Carbonic anhydrase 13 (CA13), a variant of carbonic anhydrase, catalyzes the reversible conversion of carbon dioxide to bicarbonate ions and protons. This enzymatic activity is crucial for maintaining acid–base homeostasis (Nocentini & Supuran, 2018; Supuran, 2007, 2008). CA13's role extends beyond simple carbon dioxide elimination from tissues (Alterio et al., 2006; Pastorekova et al., 2004; Thiry et al., 2006); it is particularly vital in managing acid–base balance during anaerobic glycolysis (Hilvo et al., 2008), making it an important enzyme for adaptation in high‐altitude environments. Coiled‐coil domain‐containing protein 80 (CCDC80), located in the extracellular matrix and characterized by its glycosaminoglycan‐binding activity, has emerged as a potential biomarker and therapeutic target for pulmonary arterial hypertension (PAH) (Sasagawa et al., 2016). Studies by Zhen et al. (He et al., 2023) further corroborate the relevance of CCDC80 in PAH, suggesting its pivotal role in the development of pulmonary complications during acclimatization to high‐altitude environments. DNA‐(apurinic or apyrimidinic site) lyase (APEX1) functions as a multifaceted enzyme vital for cellular homeostasis (Oliveira et al., 2022). It acts as a redox‐dependent regulator of transcription factors, including NF‐kB and HIF‐1α, which are essential in cellular hypoxia signaling, senescence, and inflammatory pathways (Laev et al., 2017). Additionally, APEX1 plays a role in inhibiting adipocyte differentiation through the regulation of lipogenic transcription factors (Lee et al., 2023), implying its involvement in the acclimatized changes in lipid metabolism observed in populations adapted to high‐altitude environments. Chordin‐like protein 2 (CHRDL2), a member of the chordin protein family, primarily functions as a secreted protein with cysteine pre‐collagen repeat structural domains. It interacts with members of the transforming growth factor β superfamily, serving as a negative regulator of cartilage formation (Chou et al., 2015). While its role in cellular hypoxia is currently under‐reported, its potential significance in hypoxic conditions merits further exploration. The low‐density lipoprotein (LDL) receptor‐related protein (LRP) family, crucial for cholesterol homeostasis (Gan et al., 2020), includes LRP11, a member expressed in stress‐related brain regions such as the amygdala and hippocampus (Xu et al., 2014). The down‐regulation of LRP11 in acclimatized individuals suggests its association with cholesterol metabolism and the body's response to hypoxic stress in high‐altitude environments.

Sclerostin (SOST) is a protein primarily secreted by osteoblasts (McClung, 2017), playing a pivotal role in bone metabolism. It functions as a negative regulator of bone formation, principally by inhibiting the classical Wnt signaling pathway in osteoblasts (Robling & Bonewald, 2020). Intriguingly, under hypoxic conditions, hypoxia‐inducible factor 1‐alpha (HIF‐1α) directly interacts with the SOST gene's promoter sequence. This interaction leads to a downregulation of SOST expression, thereby enhancing the Wnt/β‐catenin signaling pathway (Li et al., 2023), (Stegen et al., 2018). The research conducted by Genetos et al. (2010) has further emphasized this phenomenon, demonstrating that hypoxia in conditions with 1% oxygen concentration significantly down‐regulates SOST transcription and protein expression while concurrently up‐regulating the Wnt signaling pathway and activating β‐catenin. These findings have profound implications for understanding bone metabolism under hypoxic conditions, such as those experienced at high altitudes.

Moreover, the present study's correlation analysis between DEPs and clinical characteristics of the migrated plateau population aligns with these findings. It confirmed the down‐regulation of SOST expression, which positively correlates with arterial SaO_2_ and negatively with Hb and HCT (Viaene et al., 2013). This correlation may suggest a crucial link between SOST and the regulation of osteogenesis under hypoxic conditions, a significant aspect of the body's adaptation to high‐altitude environments. Syndecan‐4 (SDC4), a member of the Syndecan family, serves as a major cell‐surface acetyl heparin sulfate proteoglycan receptor (Chakravarti & Adams, 2006). Its biological roles encompass a range of cellular processes including migration, proliferation, adhesion, endocytosis, tissue repair, regeneration, cell‐substrate interactions, and matrix remodeling (Addi et al., 2020; Herum et al., 2015; Valdivia et al., 2020). Remarkably, the expression of SDC4 is known to be up‐regulated during various stress conditions such as ischemia, hypoxia, and infection (Julien et al., 2007; Ozsoy et al., 2008), indicating its adaptive response to environmental challenges. In our study, we observed that the expression of SDC4 was initially up‐regulated in the plateau group when compared to the plain group. However, as altitude increased, its expression significantly decreased in the extra‐high plateau group, suggesting a nuanced response to varying levels of hypoxic stress. It was also found that this change was negatively correlated with serum uric acid concentration, which accumulates in the body in the low oxygen environment of the plateau (Du et al., 2022), suggesting that SDC4 may be involved in purine metabolism. Such an association has been less explored in previous studies, indicating a novel area of research in the context of high‐altitude adaptation. The study of DEPs in response to hypoxia provides valuable insights into the cellular mechanisms of acclimatization. These proteins, by modulating various metabolic pathways, help the body to adapt to the challenges posed by high‐altitude environments. Although the current study has deepened the understanding of how specific proteins such as SOST and SDC4 respond to and regulate the effects of hypoxia and pointed to a correlation between SOST and Hb (Viaene et al., 2013), and SDC4 and UA (Du et al., 2022), it is interesting to note that the relationship between other DEPs identified in this study (e.g., RNASE3, SNAP23, CA13, NADK, CRKL) and the changes in Hb due to hypoxia has not been widely reported. This gap in knowledge presents an opportunity for deeper investigation into the roles of these proteins in high‐altitude acclimatization. Nonetheless, this study has its limitations. The three groups were not self‐controlled, and there were slight differences in lifestyle and work intensity between participants, which may have biased the results. Additionally, the extra‐high plateau group had relatively fewer subjects, and there was a lack of multiple‐method validation studies for the identified DEPs. These factors underscore the need for further research, incorporating larger and more diverse study populations and employing a variety of validation methods to confirm the findings and extend our understanding of protein expression changes under hypoxic conditions.

## 5 CONCLUSIONS

In this study, we utilized Olink high‐sensitivity targeted proteomics to identify serum protein profiles that are differentially expressed in relation to cellular metabolism in populations well‐acclimatized to high altitudes compared to plains populations. This approach has enabled the enrichment and identification of lower abundance serum DEPs that are challenging to detect through mass spectrometry or microarrays. The study further delves into the biological functions and mechanisms of these DEPs, particularly in relation to cellular hypoxic metabolism, as documented in existing literature. However, it should be noted that many proteins in this context remain unreported in the literature. Future work will focus on refining experimental designs, expanding sample sizes for DEP validation, and investigating the role of these proteins in the acclimatization mechanism to cellular hypoxia. This research aims to provide a theoretical foundation and identify new targets for understanding and enhancing acclimatization to high‐altitude environments.

## AUTHOR CONTRIBUTIONS

Q. S. was corresponding author and was responsible for experimental design, samples collection, and manuscript writing. J. P. and Y. D. performed proteomics assays and manuscript writing. Z. Z., T. G., L. C., K. L. and L. W. performed all calculation and data analysis. All authors read and contributed to the manuscript.

## CONFLICT OF INTEREST STATEMENT

The authors declare that the research was conducted in the absence of any commercial or financial relationships that could be construed as a potential conflict of interest.

## ETHICS STATEMENT

The study was approved by the General Hospital of the Xinjiang Military Command’s Medical Ethics Board (2023RR0302) and was conducted in accordance with the ethical principles of the Declaration of Helsinki.

## Supporting information


Table S1.

Table S2.

Table S3.

Table S4.

Table S5.

## Data Availability

The data that support the findings of this study are available from the corresponding authors on reasonable request.
